# Production of Fusaric Acid by *Fusarium* spp. in Pure Culture and in Solid Medium Co-Cultures

**DOI:** 10.3390/molecules21030370

**Published:** 2016-03-18

**Authors:** Nadine Bohni, Valérie Hofstetter, Katia Gindro, Bart Buyck, Olivier Schumpp, Samuel Bertrand, Michel Monod, Jean-Luc Wolfender

**Affiliations:** 1 School of Pharmaceutical Sciences, EPGL, University of Geneva, University of Lausanne, Quai Ernest-Ansermet 30, CH-1211 Geneva 4, Switzerland; nadine.bohni@unige.ch or nadine.bohni@chem.uzh.ch (N.B.); samuel.bertrand@unige.ch or samuel.bertrand@univ-nantes.fr (S.B.); 2 Mycology and Biotechnology Group, Institute for Plant Production Sciences IPS, Agroscope, Route de Duillier 50, P. O. Box 1012, CH-1260 Nyon, Switzerland; valerie.hofstetter@agroscope.admin.ch (V.H.); katia.gindro@agroscope.admin.ch (K.G.); olivier.schumpp@agroscope.admin.ch (O.S.); 3 Muséum National d’Histoire Naturelle, Département Systématique et Évolution, CP 39, ISYEB, UMR 7205 CNRS MNHN UPMC EPHE, 12 rue Buffon, F-75005 Paris, France; buyck@mnhn.fr; 4 Department of Dermatology and Venereology, Laboratory of Mycology, Centre Hospitalier Universitaire Vaudois, CH-1011 Lausanne, Switzerland; michel.monod@chuv.ch

**Keywords:** multi-locus phylogenetic analyses, microorganism co-culture, solid medium, induction, fusaric acid, confrontation zone, UHPLC-TOFMS

## Abstract

The ability of fungi isolated from nails of patients suffering from onychomycosis to induce *de novo* production of bioactive compounds in co-culture was examined. Comparison between the metabolite profiles produced by *Sarocladium strictum*, by *Fusarium oxysporum*, and by these two species in co-culture revealed *de novo* induction of fusaric acid based on HRMS. Structure confirmation of this toxin, using sensitive microflow NMR, required only three 9-cm Petri dishes of fungal culture. A targeted metabolomics study based on UHPLC-HRMS confirmed that the production of fusaric acid was strain-dependent. Furthermore, the detected toxin levels suggested that onychomycosis-associated fungal strains of the *F. oxysporum* and *F. fujikuroi* species complexes are much more frequently producing fusaric acid, and in higher amount, than strains of the *F. solani* species complex. *Fusarium* strains producing no significant amounts of this compound in pure culture, were shown to *de novo* produce that compound when grown in co-culture. The role of fusaric acid in fungal virulence and defense is discussed.

## 1. Introduction

Onychomycosis is a nail infection caused by systematically diverse fungi, most often by dermatophytes of the genus *Trichophyton* or, in the case of patients suffering from chronic mucocutaneous candidiasis, also by species of *Candida* [1]. However, in the case of immunodepressed people, recent studies have shown that also non-dermatophyte fungi (NDF) such as *Fusarium* spp. [2,3,4,5,6], *Acremonium* spp. [7,8] and *Aspergillus* spp. [9,10], are often reported to be the causal agents of onychomycosis. No treatment is presently efficient against these NDF pathogens.

The reasons for the occurrence of one or the other fungal pathogen remain unknown. Apart from the repeated isolation of these fungi from symptomatic nails, very little is known about the full mycobiome inhabiting infected or for that matter healthy nails. As fungi and bacteria are ubiquitous, it seems reasonable to assume that several species of fungi can inhabit a single human nail and compete to occupy this particular niche by producing secondary metabolites for defense or to access nutrients. Fungi produce a high diversity of secondary compounds and their induction is often accompanied by changes in the morphotypes of the involved species (reproduction cycles, growth inhibition or stimulation) [11]. Genomic analyses of several fungal strains suggest the potential of fungi to produce secondary metabolites is even larger than previously thought [12]. The reaction of a given strain to stimuli from the community, e.g., the activation of gene expression for the production of toxins, can ensure individual viability [13]. On the other hand, the capacity to react to external stimuli is crucial for the plasticity of a community and thus its survival [14]. The combined growth of onychomycosis-derived human pathogenic fungi together in a co-culture experiment mimics the mycobiome of onychomycosis-affected nails and might stimulate the production of antifungal compounds involved in competitive interactions. This may lead to the discovery of novel compounds with potential use in onychomycosis treatment.

The *de novo* identification of novel metabolites requires structure elucidation by NMR, a method that usually requests a significant amount of the targeted compound, thus a large quantity of living material [15]. Most reports on the isolation of microbial metabolites include large-scale liquid culturing (fermentation) [16,17,18] while solid media cultures are rarely used [19]. However, the extraction processes of solid media cultures are tedious and large-scale culturing of microorganisms is more easily done with fermentation. On the other hand, microorganisms tend to produce different compounds depending on the culture medium and method [20] and in general, extracts from solid medium-cultured fungi are more compound-rich than liquid culture extracts [21]. Therefore, strategies have been developed recently for the isolation of fungal metabolites from solid media cultures to make this field accessible to chemical metabolite analysis [22].

For metabolite profiling and dereplication by LC-HRMS, only a few micrograms of extract are needed since high quality MS and tandem MS (MS/MS) spectra can be acquired on-line with nano- to picogram-amounts of compound [15,23]. *De novo* identification of unknown metabolites—or of metabolites that are not unambiguously identifiable—requires targeted isolation for further 1D and 2D-NMR identification. This can be achieved at the microgram level with state-of-the-art microNMR methods [24].

The aim of this study was to investigate metabolomic modifications induced by co-culture of onychomycosis-related fungi, mainly *Fusarium*. Furthermore, the study is intended to showcase the targeted isolation of induced compounds derived from merely a few Petri dishes of solid medium co-cultures and produce sufficient amounts of material for NMR analysis and structure determination.

## 2. Results and Discussion

Onychomycosis-derived fungi were phylogenetically identified using five loci and several pairs of these fungi were cultured on Petri dishes and screened for morphological changes in fungal growth with the aim of finding *de novo* induced compounds. One co-culture was selected for in-depth analysis of its chemical constituents. Then, compound induction was monitored by UHPLC-TOFMS metabolite profiling. Induced metabolites were targeted for rapid isolation and for structure confirmation by NMR. In a second step, the accumulation of this particular induced compound was studied in a larger panel of pure cultures and co-cultures including both agriculturally and medically relevant strains.

Building on previous studies, experiments of induction phenomena in fungal co-cultures were done on solid medium rather than by fermentation. This has several advantages, e.g., solid media mimic the natural growth conditions of filamentous fungi in an aerobic environment and aid the development as it is a solid support. Furthermore, solid media enable the visualization of the fungal development over the experimental time and allow excision and direct analysis of the area of interest (e.g., the confrontation zone) of the agar media.

### 2.1. Fungal Identification

Out of the fungal pure cultures obtained from nail fragments of patients suffering from onychomycosis, 58 were selected for this study. Based on the top score similarity of their rDNA internal transcribed spacers (ITS) sequences with sequence(s) of GenBank, these fungi were roughly identified as dermatophytes such as *Trichophyton cf. rubrum* (Castell.) Sabour. 1911 (GenBank accession number [GBan]: KU720895) and NDF such as *Sarocladium strictum* (W. Gams) Summerbell 2011 (= *Acremonium strictum* W. Gams 1971), (GBan: KU720896), *Aspergillus* P. Micheli ex Haller 1768 spp., (GBan: KU720897, KU720898), and *Fusarium* spp. Link 1809 spp. (Table S1). Two additional agricultural *Fusarium* strains were sampled as well (see Section 3.1).

Although ITS is presently the best barcode sequence for fungi [25], it did not allow the assignment of most of the *Fusarium* isolates to a species or even to a species complex: ITS sequence BLAST top scores in GenBank were often matching sequences of undetermined *Fusarium* spp. (Table S1). Consequently, parts of four additional loci were amplified and sequenced: transcription elongation factor 1-alpha (*TEF*1-α), RNA polymerase II second largest subunit [RPB2]), β-tubulin and calmodulin (Table S1). These sequences were combined with GenBank sequences for a representative sampling of the *Fusarium* genus (47 species). *Ilyonectria radicicola* was used as the outgroup. The full combined alignment of the five loci included 104 taxa and was 3470 bp long (ITS: 548 bp, *TEF*1-α: 520 bp, *RPB*2: 1602 bp, β-tubulin: 371 bp, calmodulin: 429 bp). After removal of ambiguous regions (parts of ITS1 and 2, spliceosomal introns in protein-coding genes plus a small hyper-variable region in *RPB*2) the alignment used to perform phylogenetic analyses was of 2801 bp.

**Figure 1:**
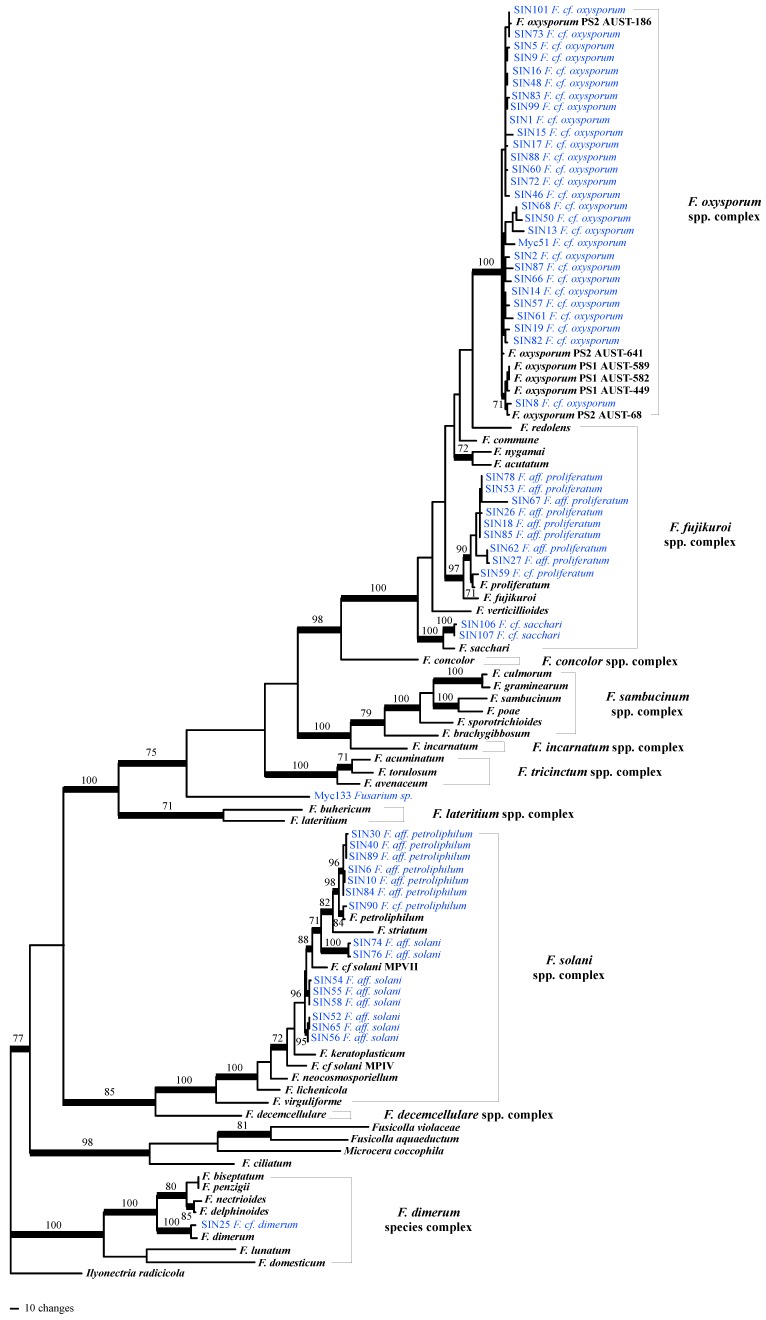
One of the most parsimonious trees (length = 4200 steps, consistency index = 0.2623, homoplasy index = 0.7373) inferred by analysis of 5-locus/104 taxa dataset. Branches that received significant bootstrap support (≥ 70%) are in bold. Strains phylogenetically analyzed for this study are highlighted blue. Abbreviations used: *F.*: *Fusarium*; *cf.* refers to taxa that were likely to be the same species, *aff.* (for affinity) refers to less closely related taxa, supported to be different from a named species, or to taxa that were not monophyletic with any named species in the *F. solani* spp. complex .

After having tested for topological incongruence and removal of conflicting sequences from the alignments (one RPB2 and two calmodulin sequences), phylogenetic analyses were performed on the combined 5-locus/104 taxa dataset. Based on the inferred tree (Figure 1), onychomycosis–associated *Fusarium* strains cluster into four *Fusarium* species complexes: in the *F. oxysporum* spp. complex (*F. cf. oxysporum*, 27 strains), in the *F. fujikuroi* spp. complex (*F. cf. proliferatum* [nine strains] and *F. cf. sacchari* [two strains]), in the *F. solani* spp. complex (with seven strains clustering with *F. petrophilum* and eight strains clustering in three different subclades that remained unresolved within the *F. solani* spp. complex based on our sampling), and in the *F. dimerum* species complex (one strain). The two *Fusarium* isolated from plants were identified as *F. cf. oxysporum* (Myc51) and as an unknown *Fusarium* sp. (only 97% of sequence similarity BLAST top score with Genbank sequences; Table S1).

### 2.2. Choice of Fungal Co-Culture for Metabolite Profiling

To simulate interaction that are likely to occur in a nail microenvironment, a large number of the above phylogenetically identified human pathogenic NDF (mainly *Fusarium* spp.) were co-cultured with one partner. *Sarocladium cf. strictum* SIN29 was selected as a partner because of its diverse metabolite composition and fast growth. Indeed, metabolite profiling (see Section 2.3) of its pure culture revealed its capacity to produce chlorinated compounds based on the observed typical isotopic distribution pattern in the MS spectra (Δ 2 Da between [M] and [M + 2] isotopes with relative intensity 3:1). This strain grows rapidly when cultured on the solid medium potato dextrose agar (PDA), as it is able to cover a 9-cm diameter Petri dish within 1–2 weeks at room temperature. Co-cultures were done on PDA in Petri dishes and the confrontation behavior of the fungi was assessed visually. The observed confrontation types included overgrowth, contact inhibition and distance inhibition ([26], Table S4). All co‑cultured fungi for which a contact or distance inhibition could be observed, grew more slowly (growth inhibition) compared to their growth as a pure culture. This observation might be explained by the production of volatile or water-diffusible antifungal compounds by one or both strains that, in consequence, results in reduced growth [27].

**Figure 2:**
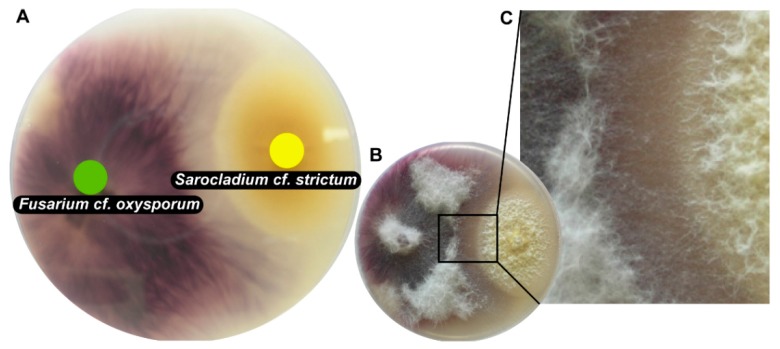
Picture of co-culture of *Fusarium cf. oxysporum* SIN17 and *Sarocladium cf. strictum* SIN29 grown on solid medium in 9-cm Petri dishes. (**A**) shows the bottom view of the co-culture with *F. cf. oxysporum* on the left (green dot) and *S. cf. strictum* to the right (yellow dot); (**B**) shows the top view of the co-culture; (**C**) shows a close-up view of the altered mycelial growth in the interaction zone between the two fungi.

The majority of tested fungi grew fast and were able to cover a 9-cm Petri dish within 1 to 2 weeks at room temperature. Among these fast-growing fungi that were confronted with *S. cf. strictum* SIN29, the following co-cultures were estimated to be of particular interest for further investigation based on their macroscopic appearance. For co-cultures involving *S. cf. strictum* SIN29 and *Fusarium cf. oxysporum* SIN2, the *Aspergillus cf. sydowii* SIN22 and *Aspergillus unguis* SIN31, a distance inhibition was observed. This confrontation pattern suggested the release of antifungal substances in the medium. This co-culture “phenotype” was thus considered interesting for the search for antifungal compounds [28,29]. Several strains showed particularly clear contact inhibition; for the co-culture with *F. cf. oxysporum* SIN17 (Figure 2), it could be observed that hyphae of both strains were in contact but mycelial morphology was altered in the confrontation zone (Figure 2C). This co-culture, *S. cf. strictum* SIN29 with *F. cf. oxysporum* SIN17, was chosen for an in-depth chemical analysis. Both strains were derived from nails of patients with onychomycosis that were resistant to standard treatment (azole drugs, such as terbinafine and itraconazole).

### 2.3. Metabolite Profiling and Automated Dereplication of Fungal Pure Culture and Co-Culture Extracts

**Figure 3:**
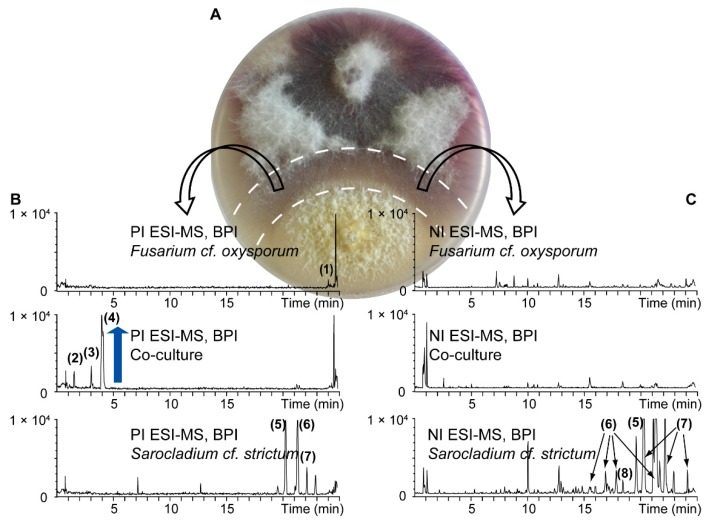
UHPLC-TOFMS metabolite profiling of pure cultures of *F. cf. oxysporum* and *S. cf. strictum* and their co-culture. For the co-culture extract, the confrontation zone ((**A**) excised zone indicated by dashed line) was cut to get an extract of the interaction zone. Base peak ion (BPI) chromatograms (**B**) in positive ionization (PI) and (**C**) negative ionization (NI) mode are shown. Metabolite induction in the co-culture is highlighted by a blue arrow. Numbered peaks were putatively assigned to known fungal metabolites based on a dereplication procedure detailed in the Supplementary Materials. The assigned compound identity is given in Tables S2 and S3.

For the chemical investigation of secondary metabolites, pure cultures and a co-culture of *S. cf. strictum* and *F. cf. oxysporum* were prepared. For the co-culture, to get a detailed picture of the metabolite production in the interaction area, the central confrontation zone was carefully excised (Figure 3A) to obtain the co‑culture extract. These three extracts were analyzed by UHPLC-TOFMS in both positive (PI) and negative ionization (NI) mode (Figure 3B,C).

Overall, for this particular set of extracts, more compounds were detected in the NI mode, especially for the pure culture extracts. For the *S. cf. strictum* pure culture extract, all the detected compounds in PI were also detectable in NI but the extract constituents were better ionized in NI which led to the detection of additional compounds. Only few compounds were detected in the co‑culture extract. This is in accordance with the fact that only the confrontation zone was extracted that comprises almost no fungal biomass but mainly fungal compounds released into the medium. Visual inspection of the co-culture chromatograms showed an intense peak for a compound in PI at 4 min (**4**, mass-to-charge ratio (*m*/*z*) 180.1030) in the co-culture extract, which was absent in both pure culture extracts. Two additional peaks, however of lower intensity, were also detected in the co-culture extract only (**2**, *m*/*z* 196.0990 at 1.5 min, **3**, *m*/*z* 178.0880 at 3.0 min). These compounds appeared *de novo* induced upon co-culture, this means, their production could not be observed in either of the pure cultures but only in the co-culture [26]. 

In addition to the annotation of induced compounds, the high-resolution chromatograms from UHPLC-TOFMS were dereplicated for known fungal metabolites reported in the Dictionary of Natural Products (DNP, version 22:1, CRC Press, Taylor & Francis, Abingdon, UK) based on an automated procedure using MZmine 2. The detailed analysis is presented as Supplementary Results.

Based on the results from HRMS and the general dereplication procedure applied to theses fungal extracts (see Supplementary Results), the molecular formula of the most intense compound (**4**, *m*/*z* 180.1030 Da) was C_10_H_13_NO_2_. This information, cross-searched with literature on *Fusarium* metabolites, strongly suggested that this compound was fusaric acid. To further confirm this hypothesis, **4** was targeted for isolation using semi-preparative HPLC (see Section 2.4).

### 2.4. Isolation of Induced Compounds from a Solid Medium Co-Culture Extract for Structure Confirmation

To ascertain the identity of the induced peaks and in order to estimate the produced amounts, targeted isolation was attempted on a limited number of Petri dishes. This procedure was also used to estimate if an NMR spectrum could already be retrieved from compounds that are largely induced in the central confrontation zone of the co-culture. It was estimated that three Petri dishes would yield sufficient sample amount for 1D-NMR analysis. (It was assumed that the induced compound makes up for 1% of the co-culture extract. The extractions for metabolite profiling (see Section 2.3) had yielded 4 mg of crude extract per Petri dish. Three Petri dishes would thus yield approx. 120 μg of pure sample.)

**Figure 4:**
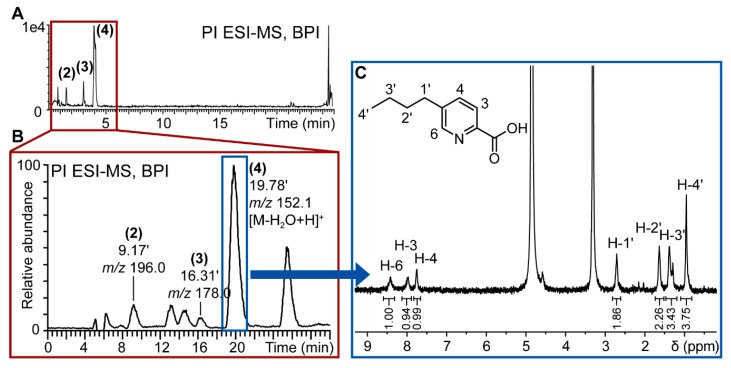
Workflow for targeted isolation of induced compounds in fungal co-culture extracts. (**A**) Base peak ion (BPI) chromatogram of positive ionization (PI) UHPLC-TOFMS metabolite profiling of *F. cf. oxysporum* and *S. cf. strictum* co-culture; (**B**) PI MS chromatogram of semi-preparative purification of *S. cf. strictum* and *F. cf. oxysporum* co‑culture extract. The targeted induced compounds eluted at 9 (**2**), 16 (**3**) and 19 (**4**) min. The chromatographic gradient had been geometrically transferred for optimal comparability of analytical and semi-preparative scale chromatography; (**C**) ^1^H-NMR spectrum of *de novo* induced compound (**4**) that corresponds to fusaric acid. Spectrum acquisitioned in CD_3_OD on CapNMR™ probe (500 MHz spectrometer, Varian Inc., Palo Alto, CA, USA; probe: Protasis/MRM, Savoy, IL, USA) at 25 °C with 128 transients. The structure of fusaric acid, the assignments and the integrals (normalized to resonance at 8.41 ppm) are given in the spectrum.

For the targeted isolation of the *de novo* induced compounds (**2**–**4**), the previously optimized gradient method used for UHPLC analysis was transferred geometrically [30] to an HPLC column with milligram amounts of loading capacity to enable the purification of extract constituents within one purification run. The semi-preparative isolation was monitored online by UV and PI electrospray ionization-MS (ESI-MS) as the target compounds were detected in PI mode only. The online MS monitoring permits the direct *m*/*z* assignment of every collected peak and allowed the direct localization of the targeted compounds (Figure 4).

The microfractions containing the targeted compounds were directly prepared for injection into the microflow NMR probe. The ^1^H-NMR spectrum of **4** is very compatible with the structure of fusaric acid (Figure 4C) which was confirmed by comparison with a reference spectrum.

The quantity of isolated compound was too low to be accurately weighed. As the peak area of the signals in ^1^H-NMR is proportional to the amount of sample, the compound amount was estimated based on a comparison of the peak area of the residual protonated solvent signal to the peak area of a proton of the analyzed compound. The isolated quantity of fusaric acid could thus be estimated to be of approx. 100 μg. In the confrontation zone, this would correspond to a concentration of almost 4 ppm, which has been reported to be toxic for plant pathogenic microorganisms [31].

The other two induced compounds **2**, **3** were collected as well, but their quantity was beyond the detection limit of our NMR instrumentation (estimated to 10 μg), even for the acquisition of a 1D-, ^1^H-NMR spectrum. Only three 9-cm Petri dishes were used for the isolation of the main induced compound **4**. Thus, in selected cases, it is feasible to isolate major induced fungal metabolites from solid medium co-cultures at a small scale to obtain exploitable NMR spectra. The limitation of the approach, however, is strongly linked to the sensitivity of the NMR instrumentation used for spectral acquisition. In our case, 1D proton sensitivity was estimated to approx. 10 μg, but state-of-the-art systems can have lower detection limits and demanding ^1^H-, ^13^C-, 2D-NMR spectra can be exploited with less than 10 μg [32].

### 2.5. Strain-Dependent Production of Fusaric Acid and Inducibility through Fungal Interaction

Fusaric acid was reported in 1934 by Japanese researchers as a secondary metabolite of *Gibberella fujikuroi* (Sawada) Wollenw. (current name *Fusarium monoliforme*). Since then, it has been reported to occur in many subspecies of the genus *Fusarium* [33]. Studies by Bacon *et al.* [34] have shown that the amount of fusaric acid produced by agriculturally relevant *Fusarium* spp. was strain- and growth medium-dependent.

Based on biosynthetic considerations, the producer of fusaric acid in the co-culture is likely *F. cf. oxysporum*. While this is well known for agricultural strains, fusaric acid production has not been shown for human-derived *Fusarium* spp. Furthermore, it appears probable that the production of fusaric acid is not only inducible upon change of culture medium but also by co-culture with another fungus. Hence, this is the first example of the induction of the mycoalexin fusaric acid through fungal interaction, by physical contact, by response to fungal metabolites or both.

To cement the finding that the production of fusaric acid in *Fusarium* spp. is strain-dependent and its production can be induced by co-culture, this biomarker was systematically searched in a large set of fungal strains using targeted metabolomics based on UHPLC-TOFMS analyses. These strains were isolated from nails of onychomycosis-patients, as plant pathogens or as environmental saprophytes [26].

**Figure 5:**
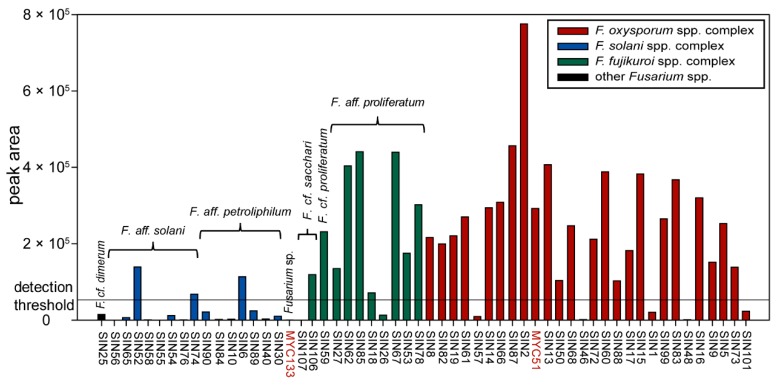
Prevalence of fusaric acid in *Fusarium* pure culture extracts. Data are grouped according to the Fusarium clades identified by phylogenetic analysis (see Figure 1). Peak area values beyond <50,000 (detection threshold adopted here) are considered as noise. All strains are of human origin, except MYC51 and MYC133 (highlighted in red).

Among all available fungal pure culture extracts, 56 were of the genus *Fusarium*. Thereof, 35 strains produced fusaric acid when grown in Petri dishes on PDA (Figure 5). Producer strains cluster in different *Fusarium* spp. complexes (three strains in the *F. solani* spp. complex corresponding to three different species in our phylogeny (see Figure 1); nine strains in *F. fujikuroi* spp. complex, corresponding to three different species; 23 strains in the *F. oxysporum* spp. complex that might correspond to two different species (*i.e.*, SIN8 which is supported (BS = 71%) to be part of a different clade than the other sampled isolates)). The amount of fusaric acid produced by individual *Fusarium* strains appears also highly variable between strains of the same species (see Figure 1 and Figure 5). This result is similar to the findings of Bacon *et al.* [34] who had shown that the amount of fusaric acid produced by agriculturally relevant *Fusarium* spp. was strain-specific. However, this study shows that the high majority of strains producing a significant amount of fusaric acid belong to the *F. fujikuroi* and *F. oxysporum* spp. complexes (Figure 5: 32 out of the 35 strains producing fusaric acid), but also that the strains of these two species complexes produce the highest amounts of this compound (Figure 5: 26 strains within these two *Fusarium* spp. complexes produce more fusaric acid than all the strains of the *F. solani* spp. complex). Furthermore, under the given culture conditions, not only environmental [34] but also human-derived *Fusarium* spp. produce fusaric acid. Whether this toxin is produced in nails and the possible biological implications this might entail for onychomycosis remains to be determined. Under the experimental conditions of the screening, *F. cf. oxysporum* SIN17 produced fusaric acid in pure culture without the stimulus of a co-culture. This could possibly be due to the differences in growth conditions (temperature and harvest time) and a time-dependent metabolite production which was has been evidenced for other co-cultures already [35,36].

**Figure 6:**
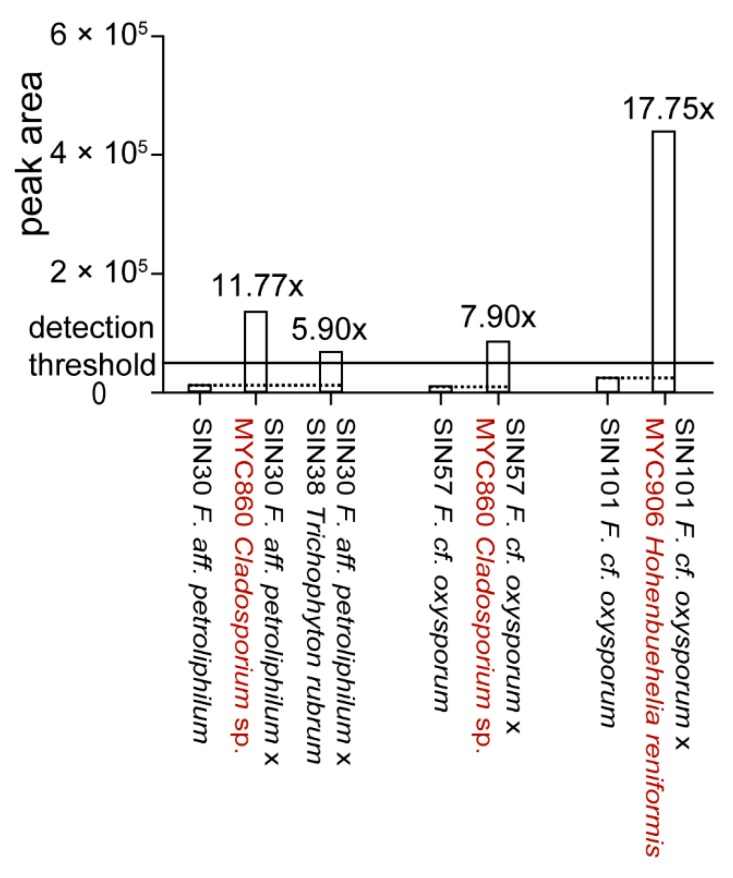
*De novo* induction of fusaric acid upon co-culture. Estimation of fold change variations was calculated based on extracted peak area of fusaric acid for the UHPLC-TOFMS metabolite profiles. Co-cultures in which fusaric acid production is induced are shown in comparison to fusaric acid production in the respective pure culture. Co‑cultures showing upregulation are shown in Supplementary Figure S2. All strains are of human origin, except MYC860 and MYC906 (highlighted in red).

To determine if *Fusarium* strains that did not produce significant amounts of fusaric acid in pure culture were able to produce it in co-culture, 106 co-cultures including *Fusarium* spp. [26] were analyzed for fusaric acid production. *De novo* induction of fusaric acid could be observed in four co-cultures (Figure 6).

The upregulation of fusaric acid production (>2x) upon co-culture could be observed in 12 co‑cultures (Figure S2). More precisely, four fungi led to an upregulation in some *Fusarium* spp. from 2.8 to 18 times. For the majority of *Fusarium* spp., the production of fusaric acid was lower upon co-culture with a second fungus (data not shown). A ratio of around 0.5 (14 co-cultures) can be explained due to a dilution effect as two fungal metabolomes are present in the extract and might be interpreted as a non-affected production. Substantial lower ratios (0.1 and less) were observed for 36 co-cultures. This can be putatively explained by detoxification of fusaric acid [37].

## 3. Experimental Section

### 3.1. Taxon Sampling and Phylogenetic Analyses

More than a hundred fungal pure cultures were obtained from nails fragments of patients suffering onychomycosis at the Centre Hospitalier Cantonal Vaudois (CHUV, Lausanne, Switzerland) [38], from plants and soil. Pure cultures and co-cultures were prepared as previously described [19] and growth conditions are detailed in the Supplementary Experimental Section. The strains were identified based on macromorphological traits and comparison of ITS sequence. These fungal strains were first roughly identified based on the top score similarity of their ribosomal internal transcribed spacers ITS1-5.8S-ITS2 nucleotide sequence (ITS) with GenBank sequence(s) (nucleotide BLAST search excluding uncultured/environmental sample sequences). Two additional *Fusarium* strains were sampled from Mycoscope [39] that were isolated from plants. All strains are stored at Mycoscope in long term preservation vials containing diluted potato dextrose (PD, BD Difco, Sparks, MD, USA) broth solution (1:4) at 4 °C. 

For *Fusarium* strains, four more loci were sequenced and a multi-locus phylogenetic analyses was performed. Part of the transcription elongation factor 1-alpha (*TEF*1-α) using primers *EF*1-1F and *EF*1-1R [40], part of the RNA polymerase II second largest subunit [*RPB*2]) using primers f*RPB*2-5F and f*RPB*2-7cR for region 5-7 and f*RPB*2-7cF and f*RPB*2-11aR for region 7-11 [41], part of β-tubulin using primers Bt2a and Bt2b [42], and part of calmodulin with primers CAL-228F and CAL-737R [43] (Table S1) were amplified and sequenced. Amplification of these loci used reagents and conditions of the Taq PCR core kit (Qiagen Inc., Valencia, CA, USA). Sequencing was performed with the amplification primers, reagents and conditions of the BigDye^®^ Terminator v3.1 Cycle sequencing Kit (Perkin Elmer, Applied Biosystems, Foster City, CA, USA) and an automated capillary sequencer ABI 3700 DNA analyzer (Perkin Elmer, Applied Biosystems). Sequences were assembled, corrected and edited in the software package Sequencher 3.0 (Gene Codes Corp., Ann Arbor, MI, USA).

For phylogenetic analyses, sequence data for 47 *Fusarium* species representative of the *Fusarium* genus in GenBank from previously published studies [3,44,45,46,47,48,49,50,51,52] was sampled and *Ilyonectria radicicola* (Gerlach & L. Nilsson) [53] was used as the outgroup. This data was combined with the *Fusarium* data. Alignments of nucleotide sequences were performed manually using the editor of MacClade v.4.06 (Maddison and Maddison, Sinauer Associates, Inc., Sunderland, MA, USA).

Topological incongruence was examined based on maximum parsimony (MP) bootstrap (BS) analyses conduced on each individual locus in PAUP* (Swofford, 2002. Sinauer Associates, Inc.). MPBS analyses included 500 BS replicates, each including 10 heuristic searches and using random addition sequence (RAS) with tree bisection-reconnection branch swapping, characters of type unordered, multistate taxa interpreted as uncertainty, one tree held at each step during stepwise addition, steepest descent option not in effect, branches collapsed if minimum branch length were zero, MAXTREES = 500 per RAS, and MULPARS option in effect. Topological conflict among trees was considered significant when different relationships for the same fungal isolates were inferred with significant support (bootstrap proportions ≥ 70%; [54,55] by the different datasets. Searches for the best MP tree(s) used 500 RAS with the same settings as for BS analyses except for MAXTREES that was limited to 1000 trees per RAS.

### 3.2. Visual Assessment of Co-Cultures

Fungal co-cultures were assessed macro- and microscopically using the following parameters: growth speed, growth margin, mycelial morphology, presence of aerial mycelia, size of hyphae, coloration of hyphae, production of liquid secretion (color), spore production (sporulation, conidiophores and conidia), spore size, presence of particular structures such as chlamydospores, color of colony, and color of medium.

### 3.3. Extraction Procedure

Fungal material and solid medium were cut in small pieces with a scalpel, citric acid-disodium hydrogen phosphate buffer (C_6_H_8_O_7_·H_2_O, 0.1 M Na_2_HPO_4_, pH 5.5) was added (twice the volume of buffer to fungal material, e.g., 20 mL of buffer solution for 10 g of fungal material) and everything mixed for 1 min (PRIMAX, Möller + Krempel AG, Bülach, Switzerland). The mixture was transferred to Erlenmeyer flasks and macerated for 4 h at 5 °C in the dark under agitation. The slurry was transferred to centrifugation bottles and centrifuged for 15 min at 4 °C with 4200 rpm (Avanti™, Beckmann Coulter™, Nyon, Switzerland). The supernatant contains mainly peptides and proteins and was not analyzed further. The pellet was washed with deionized water (centrifugation for 15 min at 4 °C with 4200 rpm) and the liquid discarded. The remaining pellet (metabolite fraction) was lyophilized. The metabolite fraction was solubilized in a monophasic solvent mixture (chloroform:methanol:water, 64:36:8 (*v*/*v*), approx. 50 mL of solvent per 1 g of lyophilized metabolite fraction, enough to obtain a suspension) and macerated for 2 h at room temperature under agitation. The extract was filtered over filter paper, washed with the solvent mixture and evaporated to dryness using a rotary evaporator. One Petri dish yielded 4 mg of crude extract.

### 3.4. Metabolite Profiling by UHPLC-TOFMS

For high-resolution metabolite profiling, the fungal extracts were purified by solid phase extraction (SPE) on C18 silica gel (Sep-Pak^®^ C18 1cc Vac cartridge, 105 μm particle size, Waters, Montreux-Chailly, Switzerland) using 80% methanol (HPLC grade, Sigma Aldrich, Buchs SG, Switzerland).

The samples (two pure culture extracts, co-culture extract and blank sample from SPE) were diluted to 1 mg/mL with 80% methanol and analyzed using UHPLC-TOFMS (Waters) in NI and PI mode in the same experiment. The analyses were performed on a Micromass-LCT Premier time-of-flight mass spectrometer equipped with an ESI interface coupled to an Acquity UPLC system. Instrumental parameters are given in Supplementary Experimental Section.

Base peak ion (BPI) chromatograms of the two pure culture and the co-culture analysis were used to assess metabolite induction. The molecular mass of compounds present in the co-culture only was searched in pure cultures using extracted ion chromatograms (XIC) to affirm metabolite induction.

### 3.5. Dereplication

The dereplication procedure was based on a previously described method [56] and is explained in detail in the Supplementary Experimental Section.

### 3.6. Semi-Preparative HPLC-MS Purification of Induced Compound

For the targeted isolation of the induced compound detected by metabolite profiling, the gradient used for metabolite profiling was geometrically transferred using HPLC Calculator 3.0 software [57] to a semi-preparative column (XTerra^®^ C18, 150 × 19 mm, 5 μm, Waters). The same solvent system as for metabolite profiling (Supplementary Experimental Section) was used. Solvent A, 0.1% formic acid (FA) in water, solvent B, 0.1% FA in acetonitrile. The compound of interest eluted within the first gradient step of the chromatogram (5%–40% B) and thus, the gradient was shortened to include only the first part of the gradient and the washing step.

The crude co-culture extract (12 mg) was solubilized in 80% methanol (0.23 mL) and filtered over a 0.45 μm Acrodisc^®^ Nylon syringe filter (BGB Analytik, Böckten, Switzerland). The purification was performed on a modular HPLC system (Varian Inc., Palo Alto, CA, USA) including UV (2151 variable wavelength monitor, LKB Pharmacia, Bromma, Sweden) and ESI-MS (LCQ, Finnigan MAT, San Jose, CA, USA) detection. Instrument parameters are given in the Supplementary Experimental Section.

Fractionation of the crude extract was performed on a 150 mm × 19 mm i.d., 5 μm, XTerra^®^ Prep MS C18 ODB™ column (Waters) at 8.0 mL/min. The injection volume was 200 μL and the transferred conditions were a gradient increasing from 5% to 40% B in 32 min followed by an increase to 100% B in 0.1 min. The column was then washed for 17 min with 100% B. Fractions were collected in tubes every minute during the first 38 min and dried on a vacuum centrifuge (Savant™ SpeedVac™, Wohlen, Switzerland).

### 3.7. Microflow NMR Analysis of the Isolated Compound

Microflow NMR analyses were done as previously described [19]. Details are given in the Supplementary Experimental Section.

### 3.8. Targeted Detection of Fusaric Acid

For automated and targeted analysis of fusaric acid prevalence in a previously recorded dataset of 229 chromatograms of pure cultures and co-cultures [26], MZmine 2 (version 2.10 [58]) and its “targeted peak detection” function was used. Therefore, the native MassLynx files from UHPLC-TOFMS analyses were transferred to NetCDF using Databridge (Waters) and imported to MZmine 2. Only chromatograms in PI mode were processed to generate peak lists. The procedure is detailed in Supplementary Experimental Section.

## 4. Conclusions

In summary, this study has proven that, in selected cases, strongly induced mycoalexins can be isolated, identified and quantified from only a very limited number of co-cultures grown on solid medium 9-cm Petri dishes. The later NMR analysis of induced metabolites is indispensable for unambiguous identification but also for a direct quantitative estimation of their concentration in the confrontation zone. With even more sensitive NMR detection methods (e.g., with microtube technology based on cryogenically cooled-NMR probes) such an approach will be even more efficient and resent a key complement to HRMS profiling.

Co-culturing *F. cf. oxysporum* SIN17 and *S. cf. strictum* SIN29, a strong induction of fusaric acid was observed. The monitoring of this compound over a large number of strains confirmed that the production of fusaric acid is strain-dependent and its induction is not always observed in co-culture. Although strain-specific, fusaric acid is overall produced, and in the highest amounts, by strains of the *F. oxysporum*–*F. fujikuroi* spp. complexes and not by strains of the *F. solani* spp. complex. However, the production of this mycoalexin can be strongly induced by interaction with a second fungal strain, this for strains that were not producing a significant amount of fusaric acid in pure culture. 

The exact mechanism that triggers such a metabolic response remains to be studied in more depth to understand if specific conditions are needed for the upregulation of this mycotoxin. Recently, it was found that trace amounts of metal (Cu^2+^ and Zn^2+^) suppress fusaric acid production when the fungus (here *F. oxysporum* f. sp. *ciceri*) was cultured in liquid medium [59]. Furthermore, a gene (FUBT) was identified that suppressed the secretion of fusaric acid into the culture medium in *F. oxysporum* f. sp. *vasifectum* [33,60]. On the other hand, culture media that were complemented with the allelochemical *p*‑hydroxybenzoic acid stimulated fusaric acid production in the plant-pathogenic *F. oxysporum* f. sp. *niveum* up to 380% [61]. The upregulation of fusaric acid production in certain *Fusarium* spp. through co-inhabiting fungi might indicate that, on top of nutrient composition, the mycobiome plays a role in virulence of *Fusarium* spp. as well. From a molecular biology point of view, gene clusters for fusaric acid biosynthesis (FUB) were detected in many *Fusarium* sspp. [33]. Examination on the presence of the FUB gene cluster in non-producing strains would pave the way for further investigation on the stimuli responsible for the strong induction of this mycoalexin in the *Fusarium* spp. highlighted here. Furthermore, as fusaric acid is often found in *Fusaria*, its use as a marker for nail fusariosis might enable new diagnostic options in onychomycosis as recently studied for *Trichophyton*-specific proteins [62].

While agriculturally pathogenic *Fusarium* spp. with their toxins and virulence factors are exhaustively studied, little research has been done on human pathogenic *Fusaria* regarding their metabolome and its implication on virulence and infection rate. In agriculturally relevant *Fusarium* pathogens, fusaric acid is described as virulence factor [59,63,64,65]. Furthermore, studies show that fusaric acid potentiates the biological activity of other mycotoxins in rodents [66]. However, it remains to be studied whether this mycotoxin is also produced in the nail during infection. It would be interesting to examine the relation and regulation of co-inhabiting fungi on fusaric acid accumulation as well as its correlation with infection rate and treatment outcome.

## Supplementary Materials

Supplementary materials can be accessed at: http://www.mdpi.com/1420-3049/21/3/370/s1.
