# Supplementary material for: Production of Fusaric Acid by Fusarium spp. in Pure Culture and in Solid Medium Co-Cultures
Source: Molecules. 2016 Mar 18;21(3):370. doi: 10.3390/molecules21030370 (PMC6274276; doi:10.3390/molecules21030370)
Supplement: Supplementary file 1 [file molecules-21-00370-s001.pdf]

Supplementary Materials: Production of Fusaric Acid by *Fusarium* spp. in Pure Culture and Solid Medium Co-Cultures

Nadine Bohni, Valérie Hofstetter, Katia Gindro, Bart Buyck, Olivier Schumpp, Samuel Bertrand, Michel Monod and Jean-Luc Wolfender

1. Supplementary Results

1.1. Fusarium Voucher Table

Table S1. *Fusarium* sampling, GenBank accession numbers for the loci sequenced and GenBank BLAST top score sequence similarity.

Collection Source	ITS	GenBank BLAST Top Score Sequence Similarity	*RPB*2	*TEF*-1	β-Tubulin	Calmodulin
SIN 1	KU720839	*F.* sp., KT351600, 100%	KU720899	KU720951	KU720743	KU720799
SIN 2	KU720840	*F.* sp., KT351600, 100%	KU720900	KU720952	KU720744	KU720800
SIN 5	KU720841	*F.* sp., KT351600, 99%	KU720901	KU720953	KU720745	KU720801
SIN 6	KU720842	*F.* sp., AF178404 100%	KU720902	KU720993	KU720746	KU720828
SIN 8	KU720843	*F. oxysporum*, GU566301, 100%	KU720903	KU720954	KU720747	KU720802
SIN 9	KU720844	*F.* sp., KT369822, 100%	KU720904	KU720974	KU720748	KU720825
SIN 10	KU720845	*F.* sp., AF178404, 99%	KU720905	KU720994	KU720749	KU720829
SIN 13	KU720846	*F.* sp., KT369822, 100%	KU720906	NO	KU720750	KU720803
SIN 14	KU720847	*F.* sp., KT369822, 100%	KU720907	KU720955	KU720751	KU720804
SIN 15	KU720848	*F.* sp., KT369822, 100%	KU720908	KU720956	KU720752	KU720805
SIN 16	KU720849	*F.* sp., KT369822, 100%	KU720909	KU720957	KU720753	KU720806
SIN 17	KU720850	*F.* sp., KT369822, 100%	KU720910	KU720958	KU720754	KU720807
SIN 18	KU720851	*F. prolif.*, KM231816, 100%	KU720911	KU720979	KU720755	NO
SIN 19	KU720852	*F. oxysporum*, KP267174 100%	KU720912	KU720959	KU720756	KU720808
SIN 25	KU720853	*F. dimerum*, EU926260, 100%	KU720913	KU720950	KU720757	KU720798
SIN 26	KU720854	*G. moniliformis*, JF499683, 100%	KU720914	KU720977	KU720758	NO
SIN 27	KU720855	*F. prolif.*, KM231816 100%	KU720915	NO	KU720759	KU720838
SIN 30	KU720856	*F.* sp., AF178404, 100%	KU720916	NO	KU720760	NO
SIN 40	KU720857	*F.* sp., AF178404, 99%	KU720917	NO	KU720761	NO
SIN 46	KU720858	*F.* sp., KT369822, 100%	KU720918	KU720960	KU720762	KU720809
SIN 48	KU720859	*F.* sp., KT369822, 100%	KU720919	KU720961	KU720763	KU720810
SIN 50	KU720860	*F.* sp., KT369822, 100%	KU720925	NO	KU720764	KU720818
SIN 52	KU720861	*F.* sp., KC808235, 100%	NO	KU720984	KU720765	NO
SIN 53	KU720862	*F. prolif.*, KM231816 100%	KU720920	KU720983	KU720766	NO
SIN 54	KU720863	*F. kerato.*, KF255446, 100%	NO	KU720985	KU720767	KU720835
SIN 55	KU720864	*F. kerato.*, KF255446, 100%	NO	KU720986	KU720768	KU720836
SIN 56	KU720865	*F.* sp., KC808235, 100%	NO	KU720987	KU720769	KU720833
SIN 57	KU720866	*F.* sp., KT369822, 100%	KU720926	KU720969	KU720770	KU720819
SIN 58	KU720867	*F. kerato.*, KF255446, 100%	NO	KU720988	KU720771	KU720837
SIN 59	KU720868	*F.* sp., EU977213, 100%	KU720927	KU720980	KU720772	NO
SIN 60	KU720869	*F.* sp., KT369822, 100%	KU720928	NO	KU720773	KU720820
SIN 61	KU720870	*F.* sp., KT369822, 100%	KU720929	KU720970	KU720774	KU720821
SIN 62	KU720871	*F. prolif.* KM231816, 100%	KU720930	KU720981	NO	NO
SIN 65	KU720872	*F.* sp., GU170639, 100%	NO	KU720989	KU720775	KU720834
SIN 66	KU720873	*F. oxysporum*, KP267174, 100%	KU720921	KU720963	KU720776	KU720812
SIN 67	KU720874	*F. prolif.* KM231816, 100%	KU720931	NO	NO	NO
SIN 68	KU720875	*F.* sp., KT369822, 100%	KU720922	KU720964	KU720777	KU720813
SIN 72	KU720876	*F.* sp., KT369822, 100%	KU720932	KU720971	KU720778	KU720822
SIN 73	KU720877	*F.* sp., KT369822, 100%	KU720933	KU720972	KU720779	KU720823
SIN 74	KU720878	*F.* sp., KP091293, 99%	KU720934	KU720990	KU720780	KU720831
SIN 76	KU720879	*F.* sp., KP091293, 99%	KU720935	KU720991	KU720781	KU720832
SIN 78	KU720880	*F. prolif.*, KM231816, 100%	KU720936	KU720978	KU720782	NO
SIN 82	KU720881	*F.* sp., KT369822, 100%	KU720937	KU720965	KU720783	KU720814
SIN 83	KU720882	*F.* sp., KT369822, 100%	KU720938	KU720966	KU720784	KU720815
SIN 84	KU720883	F. sp., KP091293, 99%	KU720939	KU720995	KU720785	NO
SIN 85	KU720884	*F. prolif.*, KM231816, 100%	KU720940	KU720982	KU720786	NO

Table S1. *Cont.*

Collection Source	ITS	GenBank BLAST Top Score Sequence Similarity	*RPB*2	*TEF*-1	β-Tubulin	Calmodulin
SIN 87	KU720885	*F.* sp., KT369822, 100%	KU720923	KU720967	KU720787	KU720816
SIN 88	KU720886	*F.* sp., KT369822, 100%	KU720924	KU720968	KU720788	KU720817
SIN 89	KU720887	*F.* sp., KP091293, 100%	KU720941	KU720996	KU720789	KU720830
SIN 90	KU720888	*F. petroli.*, KP132225, 100%	KU720942	KU720992	KU720790	KU720827
SIN 99	KU720889	*F.* sp., KT369822, 100%	KU720943	KU720973	KU720791	KU720824
SIN 101	KU720890	*F.* sp., KT369822, 100%	KU720944	KU720975	KU720792	KU720826
SIN 106	KU720891	*F.* sp., KT369822, 100%	KU720945	KU720976	KU720793	NO
SIN 107	KU720892	*F.* sp., KT369822, 99%	KU720946	NO	KU720794	NO
Myc133	KU720893	*F. buharicum*, U34581, 96%	KU720948	KU720949	KU720795	KU720797
Myc51	KU720894	*F. oxysporum*, KP267174, 100%	KU720947	KU720962	KU720796	KU720811

Collection source: SIN = Centre Hospitalier Cantonal Vaudois (CHUV, Lausanne Switzerland); Myc = (http://mycoscope.bcis.ch/). "NO" indicates missing data. Locus abbreviations: ITS = internal transcribed spacers 1 and 2 plus 5.8S rDNA; *RPB*2 = RNA polymerase II second largest subunit; *TEF*-1 = translation elongation factor 1-alpha. Species name abbreviations: *F. prolif.* = *F. proliferatum*; *F. kerato.* = *F. keratoplasticum*; *F. petro.* = *F. petroliphilum.*

1.2. Automated Dereplication of Pure Culture and Co-Culture Extracts

In addition to the annotation of induced compounds as described in the main text, the high-resolution chromatograms from UHPLC-TOFMS were dereplicated for known fungal metabolites reported in the Dictionary of Natural Products (DNP, version 22:1, CRC Press, Taylor & Francis, Abingdon, UK) based on an automated procedure using MZmine 2. As already apparent from the visual representation of the UHPLC-TOFMS chromatograms (Figure 2), only a handful of compounds were detected in positive ionization (PI) mode for all three extracts. The peak lists generated in MZmine 2 were cross-checked with *Fusarium* and *Acremonium* (synonym of *Sarocladium*) metabolites found in DNP. Extending the search to all microbial metabolites reported in DNP provides putative annotation for almost all detected peaks. As false positive rate can be very high, chemotaxonomic information was included in the dereplication procedure. In this way, a few peaks (Tables S2 and S3) could be putatively assigned based on the high mass accuracy and the spectral accuracy of the MS (level 3 according to the Metabolomics Standards Initiative (MSI) [1]).

In the pure culture extract of *S. cf. strictum*, the compounds ilicicolin C (**7**), acremofuranone A (**6**) and B (**5**) were annotated in the PI UHPLC-TOFMS profile, whereas ilicicolin C (**7**), antibiotic LL-Z 1272ε (**8**), acremofuranone A (**5**) and B (**6**) could be putatively assigned in negative ionization (NI) UHPLC-TOFMS profile. Similarly, the compound HA 23 (**1**) was identified in the PI analysis of the pure culture extract of *F. cf. oxysporum*. The three *de novo* induced compounds detected in the co-culture extract could be putatively assigned to fusarinolic acid (**2**), dehydrofusaric acid (**3**) and fusaric acid (**4**). The structure of all these dereplicated compounds are shown in Supplementary Figure S1. Ilicicolin C and antibiotic LL-Z 1272ε are listed in DNP as *Fusarium* metabolites but both compounds were recently isolated from a sponge-derived *Acremonium* sp. as well as from fungi of other genera [2-4].

The extracted ion traces corresponding to acremofuranone A and ilicicolin C evidenced the presence of various isomers in the *S. cf. strictum* pure culture extract. A search in DNP for the molecular formula of acremofuranone A ($C_{23}H_{29}O_5Cl$) returned three additional fungal metabolites (8′-hydroxyascochlorin [CRC code: OFL20, CAS: n/a], ascofuranone [CRC code: CKR68, CAS: 38462-04-3], chaetomugilin K [CRC code: PYM46, CAS: 1187848-03-8]). For the molecular formula of ilicicolin C ($C_{23}H_{31}O_4Cl$), DNP search returned two additional fungal metabolites (ilicicolinic acid A [CRC code: MWZ28, CAS: 152607-06-2], LL-Z 1272α epoxide [CRC code: PZN75, CAS: 1202381-02-9]). The detected compounds have not been automatically assigned to these other compounds as their biological source was unrelated to *Acremonium* spp. but these, or derivatives thereof, have been isolated from an *Acremonium* sp. [2]. Confident identification of the detected compounds would have necessitated comparison with authentic standards or subsequent isolation and NMR analysis (level 1 based on MSI). In this study, the interest was mainly related to the induced compounds and the targeted isolation for confirmatory analysis by NMR was performed only on the co-culture extract.

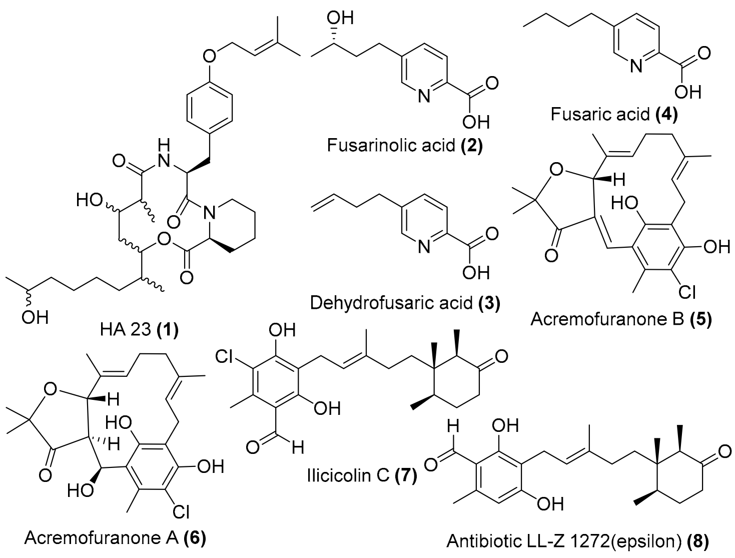

Figure S1. Structures of dereplicated molecules. The numbers correspond to its molecular formula and its occurrence in the different chromatograms is given in Figure 3 and Tables S2 and S3.

Table S2. List of detected compounds in positive ionization (PI) UHPLC-TOFMS metabolite profiling in the extracts of *F. cf. oxysporum* and *S. cf. strictum* pure cultures and the co-culture that were putatively assigned to known fungal metabolites based on the Dictionary of Natural Products (version 22:1, CRC Press, Taylor & Francis, Abingdon, UK). The derived molecular formulae are consecutively numbered and their position is shown in the chromatogram (Figure 3).

**PI UHPLC-TOFMS Metabolite Profiling**							
	***m/z* (Da)**	**Retention time (min)**	**Molecular Formula**	**Exact Mass (Da)**	**Δ (ppm)**	**CRC Code CAS**	**Putative Annotation**
F. cf. oxysporum pure culture	601.4001	24.2	$C_{34}H_{52}N_2O_7$ (**1**)	601.3853	24.6	LQX93 n/a	HA 23
co-culture	196.0990	1.5	$C_{10}H_{13}NO_3$ (**2**)	196.0974	8.2	FTS52 26108-30-5	Fusarinolic acid
	178.0880	3.0	$C_{10}H_{11}NO_2$ (**3**)	178.0868	6.7	FVX97 3626-76-4	Dehydrofusaric acid
	180.1030	3.9	$C_{10}H_{13}NO_2$ (**4**)	180.1025	2.8	FTS47 536-69-6	Fusaric acid
S. cf. strictum pure culture	403.1652	20.4	$C_{23}H_{27}O_4Cl$ (**5**)	403.1676	6.0	PVP88 1114927-91-1	Acremofuranone B
	421.1785	21.2	$C_{23}H_{29}O_5Cl$ (**6**)	421.1782	0.7	PVV28 1114927-89-7	Acremofuranone A
	407.1989	22.3	$C_{23}H_{31}O_4Cl$ (**7**)	407.1989	0.0	HJB60 1202381-02-9	Ilicicolin C

Table S3. List of detected compounds in negative ionization (NI) UHPLC-TOFMS metabolite profiling in the extracts of *F. cf. oxysporum* and *S. cf. strictum* pure cultures and the co-culture that were putatively assigned to known fungal metabolites based on the Dictionary of Natural Products (version 22:1, Taylor & Francis, Abingdon, UK). The derived molecular formulae are consecutively numbered and their position is shown in the chromatogram (Figure 2).

**NI UHPLC-TOFMS Metabolite Profiling**							
	***m/z* (Da)**	**Retention time (min)**	**Molecular Formula**	**Exact Mass (Da)**	**Δ (ppm)**	**CRC Code CAS**	**Putative Annotation**
S. cf. strictum pure culture	419.1608	15.7	$C_{23}H_{29}O_5Cl$ (**6**)	419.1625	4.1	PVV28 1114927-89-7	Acremofuranone A
	419.1607	17.0	$C_{23}H_{29}O_5Cl$ (**6**)	419.1625	4.3	PVV28 1114927-89-7	Acremofuranone A
	419.1636	17.9	$C_{23}H_{29}O_5Cl$ (**6**)	419.1625	2.6	PVV28 1114927-89-7	Acremofuranone A

Table S3. *Cont.*

NI UHPLC-TOFMS Metabolite Profiling							
	m/*z* (Da)	Retention time (min)	Molecular Formula	Exact Mass (Da)	Δ (ppm)	CRC Code CAS	Putative Annotation
S. cf. strictum pure culture	371.2205	18.5	$C_{23}H_{32}O_4$ (**8**)	371.2222	4.6	HJB61 22562-68-1	Antibiotic LL-Z 1272ε
	401.1502	20.4	$C_{23}H_{27}O_4Cl$ (**5**)	401.1520	4.5	PVP88 1114927-91-1	Acremofuranone B
	405.1860	20.6	$C_{23}H_{31}O_4Cl$ (**7**)	405.1833	6.7	HJB60 1202381-02-9	Ilicicolin C
	419.1609	21.3	$C_{23}H_{29}O_5Cl$ (**6**)	419.1625	3.8	PVV28 1114927-89-7	Acremofuranone A
	405.1805	22.3	$C_{23}H_{31}O_4Cl$ (**7**)	405.1833	6.9	HJB60 1202381-02-9	Ilicicolin C
	405.1849	24.3	$C_{23}H_{31}O_4Cl$ (**7**)	405.1833	3.9	HJB60 1202381-02-9	Ilicicolin C

2. Supplementary Experimental Section

2.1. Growth Conditions

For pure cultures, a 5-mm agar plug of a fungal preculture was inoculated in the center of a 9-cm Petri dish containing 30 mL of PDA (potato dextrose agar, Difco, 39 g per liter) prepared with distilled water. The dishes were incubated at room temperature. Co-culture experiments were inoculated with two 5-mm agar plugs of the appropriate fungal strains on opposite sides of a Petri dish containing PDA, and the dishes were incubated at room temperature until physical contact of the two fungi, usually seven days. The strains in the screening of the large set of fungi were incubated at 21 °C [5].

2.2. Metabolite Profiling by UHPLC-TOFMS

The analyses were performed on a Micromass-LCT Premier time-of-flight mass spectrometer (Waters, Montreux-Chailly, Switzerland) equipped with an evaporative ionization (ESI) interface coupled to an Acquity UPLC system (Waters, Montreux-Chailly, Switzerland). The *m*/*z* range was set to 100–1000 in centroid mode with a scan time of 0.25 s and an interscan delay of 0.01 s. The ESI conditions in the PI and NI modes were as follows: capillary voltage of 2800 V, cone voltage of 40 V, source temperature of 120 °C, desolvation temperature of 250 °C, cone-gas flow of 20 L/h, and desolvation gas flow of 600 L/h. For internal calibration, a 2 μg/mL solution of leucine-enkephalin from Sigma-Aldrich (Steinheim, Germany) was infused through the lock-mass probe at a flow rate of 10 μL/min using a second Shimadzu LC-10ADvp LC pump. The metabolite profiling was carried out on an Acquity BEH C18 150 × 2.1 mm 1.7 μm column equipped with a precolumn at a flow rate of 0.45 mL/min. The following solvent system was used. Solvent A, 0.1% formic acid (FA) in water, solvent B, 0.1% FA in acetonitrile, 7′ 5%–40% B, 16′ 40%–64% B, 1′ 64%–100% B, 8′ 100% B. The temperature was maintained at 50 °C and the injection volume was 2 μL. Solvents were ULC/MS grade (Biosolve).

2.3. Dereplication

For automated dereplication, native MassLynx data from UHPLC-TOFMS analyses were transferred to NetCDF (common data format) using Databridge (Waters, Montreux-Chailly, Switzerland). These files were then imported to MZmine 2 (version 2.10 [6]) for data processing and generation of peak lists. The chromatograms were reduced to 0.5–30 min, and masses were detected in centroid mode with a noise level of 100. Then, peak lists were built with a minimum time span per peak of 0.1 min, a minimum intensity of 1000 and an *m*/*z* tolerance of 30 ppm. The resulting peak lists were deconvoluted using the algorithm "local minimum search" and deisotoped. All chromatograms were aligned using the function "join aligner" and the combined list was filtered for duplicate peaks. The detailed parameters and the workflow are given in Table S5.

Table S4. List of fast-growing fungi co-cultured with *Sarocladium cf. strictum* SIN29 in Petri dishes and visual assessment of the confrontation behavior ([5], overgrowth, contact inhibition, distance inhibition). Fungal growth was inhibited compared to growth as pure culture for all co-cultures with contact or distance inhibition.

Overgrowth	Contact Inhibition	Distance inhibition
F. aff. petroliphilum SIN10	*F. cf. oxysporum* SIN1	*F. cf. oxysporum* SIN2
F. cf. oxysporum SIN13	unidentified strain SIN3	*Aspergillus cf. sydowii* SIN22
F. cf. oxysporum SIN14	*F. cf. oxysporum* SIN5	*Aspergillus* sp. SIN31
unidentified strain SIN20	*F. aff. petroliphilum* SIN6	
unidentified strain SIN23	unidentified strain SIN7	
F. cf. dimerium SIN25	*F. cf. oxysporum* SIN8	
F. aff. proliferatum SIN26	*F. cf. oxysporum* SIN9	
F. aff. proliferatum SIN27	unidentified strain SIN12	
F. cf. oxysporum SIN46	*F. cf. oxysporum* SIN15	
F. cf. oxysporum SIN48	*F. cf. oxysporum* SIN16	
F. cf. oxysporum SIN50	*F. cf. oxysporum* SIN17	
F. cf. oxysporum MYC51	*F. cf. oxysporum* SIN19	
F. aff. solani SIN52	*F. aff. solani* SIN54	
F. aff. proliferatum SIN53		
F. aff. solani SIN56		
F. cf. oxysporum SIN57		
F. aff. solani SIN58		
F. cf. proliferatum SIN59		
F. cf. oxysporum SIN60		
F. cf. oxysporum SIN61		
F. aff. oxysporum SIN62		

Table S5. Steps and parameters used during the automatic peak picking procedure by MZmine 2 for dereplication in metabolite profiling chromatograms. The same parameters were used for NI and PI UHPLC-TOFMS data.

Steps	Parameters		Value
(1) Raw data methods → Filtering → Data set filtering			
	Filter	Crop filter	
		Retention time (min)	0.5 to 30
(2) Raw data methods → Peak detection → Mass detection			
	Mass detector	Centroid	
		Noise Level	100
	MS level		1
(3) Raw data methods → Peak detection → Chromatogram builder			
	Min time span (min)		0.1
	Min height		1000
	m/*z* tolerance (ppm)		30
(4) Peak list methods → Peak detection → Chromatogram deconvolution			
	Algorithm	Local minimum search	
	Chromatographic threshold (%)		85
	Search minimum in RT range (min)		0.1
	Minimum relative height (%)		5
	Minimum absolute height		100
	Min ratio of peak top/edge		1
	Peak duration range (min)		0 to 10
(5) Peak list methods → Isotopes → Isotopic peaks grouper			
	m/*z* tolerance (ppm)		30
	Retention time tolerance (min)		0.5
	Maximum charge		3
	Representative isotope		Most intense
(6) Peak list methods → Alignment → Join aligner			
	m/*z* tolerance (ppm)		30
	Weight for *m*/*z*		10
	Retention time tolerance (min)		0.5

Table S5. *Cont.*

Steps	Parameters	Value
(6) Peak list methods → Alignment → Join aligner		
	Weight for RT	10
	Compare isotope pattern	
	Isotope *m*/*z* tolerance (ppm)	30
	Minimum absolute intensity	100
	Minimum score (%)	50
(7) Peak list methods → Filtering → Duplicate peak filter		
	m/*z* tolerance (ppm)	30
	RT tolerance	0.5
(8) Peak list methods → Identification → Custom database search		
	m/*z* tolerance (ppm)	30
	Retention time tolerance (absolute, min)	0.5

The detected masses within the peak lists were identified by a "custom database search" using entries of DNP (version 22:1, CRC Press, Taylor & Francis, Abingdon, UK). For this, entries with the biological source listed as *Fusarium*, *Gibberella* (name of teleomorph form of *Fusarium*) and *Acremonium* (synonym of *Sarocladium*) were exported and two CSV files were constructed that contained the CRC code as unique identifier, the compound name, the molecular formula and *m*/*z* that was calculated from the exact mass (as given in DNP) by addition or subtraction of the proton mass (1.007276 Da) for the identification of compounds analyzed in PI and NI mode, respectively. Within MZmine 2, detected compounds are identified based on their exact mass with an *m*/*z* tolerance of 30 ppm and by comparing isotopic patterns (heuristic filtering [7]) with a score of 50% or higher. The *m*/*z* tolerance and the isotopic pattern match score were chosen large to compensate for *m*/*z* errors due to alignment during data mining. Also, spectra in PI and NI mode were measured in the same chromatographic run and this diminishes the mass accuracy of the TOFMS analysis. In a second step, putatively identified compounds were searched in the original chromatograms with MassLynx (V4.1, Waters) and the molecular formula was verified using the in-built elemental composition tool (exact mass and isotopic pattern match).

2.4. Semi-Preparative HPLC-MS Purification of Induced Compound

The purification was performed on a modular HPLC system composed of a Varian 9012 pump (Palo Alto, CA, USA), a manual injection system (Rheodyne, IDEX Health & Science, Wertheim-Mondfeld Germany), a UV spectrometer set at 254 nm (2151 variable wavelength monitor, LKB Pharmacia, Bromma, Sweden) equipped with a Pharmacia LKB-Rec 1 chart recorder, a fraction collector (FC204, Gilson, Middleton, WI, USA), an adjustable flow splitter, and an MS spectrometer (LCQ, Finnigan MAT, San Jose, CA, USA) with an ESI interface. Separations were performed using water with 0.1% FA as eluent A and acetonitrile with 0.1% FA as eluent B. A fraction (1/170) of the flow was split to the MS detector for analysis. The following ESI conditions were used: capillary temperature, 180 °C; source voltage, 2.5 kV; and sheath gas nitrogen, 70 psi. The MS acquisitions were performed in PI mode using a full scan mode over an *m*/*z* range of 150–1000 and a scan time of 1 s.

2.5. Microflow NMR Analysis of the Isolated Compound

Microflow NMR analyses were performed on a Varian Unity Inova 500 MHz NMR instrument (Palo Alto, CA, USA) equipped with a 5 μL microflow NMR probe (CapNMR™) from Protasis/MRM (Savoy, IL, USA) with an active volume of 1.5 μL. The samples were dissolved in 10 μL of CD_3OD and injected manually using a microliter 25 μL syringe with a 22s gauge needle (Hamilton®). ^1H-NMR analysis was done with 128 transients at 298 K. Spectra were treated using MestReNova (version 8.0.2, Mestrelab Research S. L.) and chemical shifts were referenced to the residual protonated solvent signal (CD_3OD, 3.31 ppm).

2.6. Targeted Detection of Fusaric Acid

For automated and targeted analysis of fusaric acid prevalence in a previously recorded dataset of 229 chromatograms of pure cultures and co-cultures [5], MZmine 2 (version 2.10 [6]) and its "targeted peak detection" function was used. Therefore, the native MassLynx files from UHPLC-TOFMS analyses were transferred to NetCDF using Databridge (Waters) and imported to MZmine 2. Only chromatograms in PI mode were processed to generate peak lists.

The chromatograms were reduced to 0.5–4.5 min, and masses were detected in centroid mode with a noise level of 50. The *m*/*z* value of fusaric acid was calculated using MassLynx molecular weight calculator from molecular formula and the "targeted peak detection" function was applied with an *m*/*z* tolerance of 15 ppm (0.005 *m*/*z*). The resulting peak lists were deconvoluted using the Savitzky-Golay filter with a manually optimized derivative threshold level 90% (with the help of the preview function in MZmine 2) and deisotoped. All chromatograms were aligned using the function "join aligner", gap filled using the function "same RT and *m*/*z* range gap filler" (*m*/*z* tolerance range 15 ppm) and filtered for duplicate peaks (retention time tolerance 0.15 min). The detailed parameters are given in Supplementary Table S6. The aligned chromatograms were exported to a .csv file and further analyzed in Excel. The graphs were created with SigmaPlot (12.0, Systat Software Inc., San Jose, CA, USA).

Table S6. Steps and parameters used during the automatic peak picking procedure by MZmine 2 for targeted analysis of fusaric acid in PI UHPLC-TOFMS fingerprinting analyses.

Steps	Parameters		Value
(1) Raw data methods → Filtering → Data set filtering			
	Filter	Crop filter	
		Retention time (min)	0.5 to 4.5
(2) Raw data methods → Peak detection → Targeted peak detection			
	Intensity tolerance (%)		50
	m/*z* tolerance (ppm)		15
	Retention time tolerance (min)		0.15
(3) Peak list methods → Peak detection → Chromatogram deconvolution			
	Algorithm	Savitzky-Golay	
	Min peak height		50
	Peak duration range (min)		0 to 10
	Derivative threshold level (%)		90
(4) Peak list methods → Alignment → Join aligner			
	m/*z* tolerance (ppm)		15
	Weight for *m*/*z*		10
	Retention time tolerance (min)		0.15
	Weight for RT		10
(5) Peak list methods → Gap filing → Same RT and *m*/*z* range gap filler			
	m/*z* tolerance (ppm)		15
(6) Peak list methods → Filtering → Duplicate peak filter			
	m/*z* tolerance (ppm)		15
	RT tolerance		0.15

Original chromatograms with positive detection of fusaric acid were manually processed in MassLynx (V4.1, Waters) and the molecular formula was verified using the in-built elemental composition tool (exact mass and isotopic pattern match). This revealed that features with a peak area of <50,000 were identified as false-positive hits for fusaric acid detection, based on peak shape, accurate mass and isotopic pattern. This peak area value was thus used as detection threshold to assess fusaric acid production (Figures 5, 6 and S2).

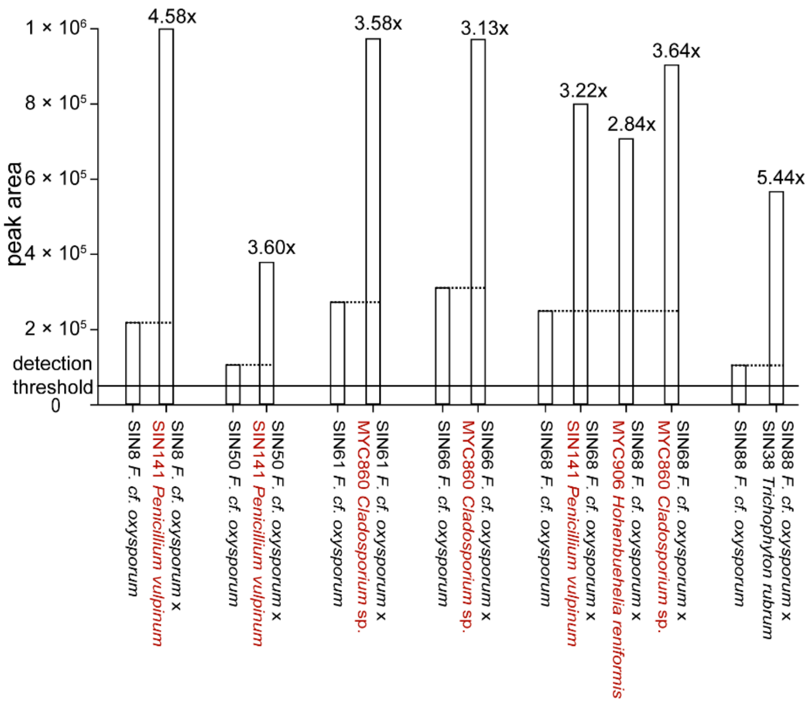

Figure S2. Upregulation of fusaric acid upon co-culture. Estimation of fold change variations was calculated based on extracted peak area of fusaric acid for the UHPLC-TOFMS metabolite profiles. Co-cultures with upregulated fusaric acid production are shown in comparison to fusaric acid production in the respective pure culture. Co-cultures where *de novo* induction was observed are shown in Figure 5 in the main text. All strains are of human origin, except SIN141, MYC860 and MYC906 (marked red).

References

1. Creek, D.J.; Dunn, W.B.; Fiehn, O.; Griffin, J.L.; Hall, R.D.; Lei, Z.; Mistrik, R.; Neumann, S.; Schymanski, E.L.; Sumner, L.W., *et al.* Metabolite identification: are you sure? And how do your peers gauge your confidence? *Metabolomics* **2014**, *10*, 350–353.
2. Zhang, P.; Bao, B.; Dang, H.T.; Hong, J.; Lee, H.J.; Yoo, E.S.; Bae, K.S.; Jung, J.H. Anti-inflammatory sesquiterpenoids from a sponge-derived fungus *Acremonium* sp. *J. Nat. Prod.* **2009**, *72*, 270–275.
3. Gutiérrez, M.; Theoduloz, C.; Rodríguez, J.; Lolas, M.; Schmeda-Hirschmann, G. Bioactive metabolites from the fungus *Nectria galligena*, the main apple canker agent in Chile. *J. Agr. Food Chem.* **2005**, *53*, 7701–7708.
4. Isaka, M.; Yangchum, A.; Supothina, S.; Laksanacharoen, P.; Jennifer Luangsa-ard, J.; Hywel-Jones, N.L. Ascochlorin derivatives from the leafhopper pathogenic fungus *Microcera* sp. BCC 17074. *J. Antibiot.* **2015**, *68*, 47–51.
5. Bertrand, S.; Schumpp, O.; Bohni, N.; Bujard, A.; Azzollini, A.; Monod, M.; Gindro, K.; Wolfender, J.-L. Detection of metabolite induction in fungal co-cultures on solid media by high-throughput differential ultra-high pressure liquid chromatography-time-of-flight mass spectrometry fingerprinting. *J. Chromatogr. A* **2013**, *1292*, 219–228.
6. Pluskal, T.; Castillo, S.; Villar-Briones, A.; Oresic, M. MZmine 2: Modular framework for processing, visualizing, and analyzing mass spectrometry-based molecular profile data. *BMC Bioinf.* **2010**, *11*, 395.
7. Kind, T.; Fiehn, O. Seven golden rules for heuristic filtering of molecular formulas obtained by accurate mass spectrometry. *BMC Bioinf.* **2007**, *8*, 105.
